# Online adaptive radiotherapy: International strategies for AI-enabled workflow efficiency and radiation therapist-led delivery for sustainable practice

**DOI:** 10.1016/j.tipsro.2026.100413

**Published:** 2026-05-25

**Authors:** Meegan Shepherd, Bethany Williams, Anna Dinkla, Brayden Geary, Sarah Barrett

**Affiliations:** a Northern Sydney Cancer Centre, Royal North Shore Hospital, St Leonards, NSW, Australia; b Monash University, Clayton, VIC, Australia; c The Royal Marsden NHS Foundation Trust, UK; d Amsterdam UMC, Location Vrije Universiteit Amsterdam, Department of Radiation Oncology, Amsterdam, Netherlands; e Cancer Center Amsterdam, Amsterdam, Netherlands; f Olivia Newton John Cancer Wellness and Research Centre, Austin Hospital, Heidelberg, Victoria, Australia; g Applied Radiation Therapy Trinity, Discipline of Radiation Therapy, Trinity College Dublin, Ireland; h Trinity St James's Cancer Institute, Trinity College Dublin, Ireland

**Keywords:** Online Adaptive Radiotherapy, ART, oART Radiation Therapists (RTT) Efficiency, Training, Credentialing, AI

## Abstract

Online adaptive radiotherapy (oART) represents a significant advancement in personalised radiation cancer treatment, offering improved daily dose to targets and optimised organ exposures with reduced toxicity. Implementation remains challenging due to resource intensiveness, workflow complexity and workforce limitations. This article presents international insights on optimising oART delivery with a focus on practice development and workforce transformation. Strategies are explored to improve workflow efficiency, integration of artificial intelligence (AI) and the role of both therapeutic radiographers and radiation therapists (RTTs) in leading adaptive workflows. Credentialing frameworks for RTTs are examined as a mechanism to support sustainable oART delivery and reduce clinician console time. The discussion synthesises practical innovations from across multiple international healthcare systems, highlighting reproducible models for efficiency, workforce training and AI integration. These insights aim to guide global efforts in scaling oART delivery through efficient and collaborative practice models that align with safe, accessible patient-centred care.

## Highlights

• Global insights on implementing online adaptive radiotherapy in practice
• RTT-led adaptive workflows improve access and treatment efficiency
• Structured credentialing supports safe, sustainable oART delivery
• AI integration streamlines imaging, contouring and plan generation
• Collaborative models enhance patient-centred adaptive care worldwide

## Introduction

Online adaptive radiotherapy (oART) has moved from concept to routine practice in many centres. Advances in magnetic resonance imaging (MRI) and cone-beam computed tomography (CBCT)-guided adaptation including artificial intelligence (AI)-assisted workflows are driving this shift. Adoption is increasing internationally across Europe, North America, Asia and the Pacific, supported by a growing evidence base that includes increased use of hypo-fractionation with reduced margins [1], [2]. This pathway enables pre-treatment modification of plans to account for inter- and infra-fraction anatomical variation. It aims to improve geometric accuracy and reduce normal tissue exposure with early evidence suggesting improvements in survival and quality of life (QoL) outcomes [3], [4], [5], [6], [7]. When implemented efficiently, oART mitigates cumulative toxicity [8] and streamlines patient pathways [9], reinforcing its value within the radiation oncology landscape [10], [11].

The widespread availability of commercial solutions has supported the adoption of oART in routine practice, with MR-guided workflows increasing steadily since 2014 [1], [2]. As clinical experience grows, indications for oART continue to expand. However, despite hypo-fractionation and mature commercial platforms, oART remains resource-intensive [12], [13]. This burden is most apparent in ‘on-couch’ treatment time, with reported session durations ranging from 15 to 70 min [14], [15], [16], [17], [18], compared with 10-15 min for conventional techniques [13].

Emerging literature suggests that selective use of oART may reduce time-related ‘toxicity’ within non-adaptive workflows [19]. Technological innovation, including AI-enabled workflows, forms part of the broader efficiency landscape and offers opportunities to streamline delivery [20]. Despite established dosimetric benefits, translation of oART into routine practice remains limited in many centres by gaps in clinical evidence and operational constraints associated with this resource-intensive model.

These challenges are further shaped by jurisdiction specific governance frameworks and variation in baseline professional training for Radiation Therapists (RTTs), a term used here to include therapeutic radiographers internationally, contributing to disparities in access between institutions and global regions [21], [22], [23]. Improving access to oART therefore requires a multi-pronged approach that addresses both clinical evidence and workflow efficiency.

This paper consolidates international perspectives and examines strategies to overcome workflow and technological related barriers, alongside consideration of how governance, credentialing models and training pathways influence the feasibility of RTT-led oART across healthcare settings.

## Workflow Efficiency in Online Adaptive Radiotherapy.

While oART toxicity data is promising, broader evidence regarding cost-effectiveness is yet to be fully established [24], [25]. Combined with substantial capital outlay and high resource demands, this presents significant barriers to implementation. Improving efficiency is therefore essential to expanding patient access to advanced treatments, particularly in the context of rising cancer burden. This section considers efficiency across both treatment regimen design, including fractionation and imaging pathways and on-couch workflow processes during adaptive delivery. Importantly this discussion focuses on countries with widespread access to established radiotherapy infrastructure, workforce capacity and access to advanced technologies, the underlying principles of efficiency and resource optimisation are relevant globally.

oART precision has facilitated increased adoption of ultra-hypofractionation, reducing margins, patient visits and improving treatment convenience without compromising clinical outcomes [25], [26], [27]. Likewise, simulation-free radiotherapy which generates treatment plans directly from diagnostic imaging or patient agnostic reference plans, may reduce the need for dedicated pre-treatment imaging, as demonstrated in MRI-guided stereotactic workflows for prostate cancer by De Leon et al and others. [4], [24], [28] These approaches, reduce patient visits and waiting times while improving patient experience, convenience and system throughput [[24], [25], [26], [27], [28], [29]]. However, whether these efficiency gains offset high resource inputs remains unclear across sites and clinical settings, particularly where simulation-free planning requires technical workarounds or introduces additional workload. In several settings, including gynaecological radiotherapy, hypofractionation remains limited, increasing reliance on workflow efficiency and technological maturity to realise the value of oART. Demonstrating cost-effectiveness compared with conventional radiotherapy therefore depends on vendor solutions that enable streamlined, integrated workflows, rather than shifting complexity to clinical teams.

Efforts to optimise resource use increasingly focus on reducing treatment duration. Shorter treatments improve patient tolerability, reduce intra-fraction motion and increase effective machine capacity [[30], [31]]. Persistent workflow bottlenecks include contouring, decision-making and plan optimisation of driven by image quality limitations, software constraints and quality assurance (QA) requirements, as highlighted in MRI-guided workflows evaluated by Lin et al. and Willigenburg et al. [[32], [33]] Some aspects of the adaptive workflow depend on vendor-driven technological advances, including improved image contrast and more reliable auto-contouring. These technologies continue to mature and have not yet translated into consistent time savings for early adopters. Additional efficiency gains can be achieved through multidisciplinary team (MDT)-based strategies, including standardised clinical protocols, margin-based trade-offs and AI-supported decision making [[11], [34], [35], [36], [37]]. Translating these approaches into sustained clinical time savings remains complex, particularly regarding implementation and QA [[24], [37], [38]].

Selective plan adaptation, guided by decision-support systems offers a targeted approach to improving efficiency. Frequent adaptation provides value only when technically feasible and clinically indicated; unnecessary adjustments offer minimal benefit [[38], [39]]. Advances in gating and intra-fraction adaptation may further improve treatment accuracy and in principle, the cost-benefit profile [40]. AI strategies are increasingly directed toward real-time volumetric motion management, including continuous dose optimisation during beam delivery [38].

With both CBCT and MRI-based oART available, aligning platform capabilities with specific disease sites is key to maximising clinical benefit and economic value within a platform-agnostic approach [41]. International collaboration and integration within clinical trials integration is essential to build the oART evidence-base and address professional unmet needs [38]. This includes cost-savings and time efficiencies achieved through more sustainable workforce models [[11], [42]] that reduce reliance on a full MDT at every fraction, as discussed in the next section.

Efficiency gains in oART are inherently constrained by the speed, design and interoperability of commercial platforms, including software, hardware and intra-fraction motion management capability. These factors are largely vendor-determined and define current clinical limits. Progress relies on scalable and customisable solutions that improve integration and reduce workflow burden within these constraints.

Efficiency must also be considered alongside quality and safety. Sustainable gains require alignment with robust QA processes and clinical oversight, ensuring that workflow improvements translate into consistent and safe clinical delivery.

## AI Integration in Online Adaptive Radiotherapy Practice.

AI performance and clinical uptake are accelerating rapidly, with growing relevance to oART, where imaging, contouring, planning and decision making occur under time pressure [[43], [44]]. In this setting, AI has the potential to contribute most when it reduces on-couch time while preserving clinical confidence, safety and accountability, particularly within imaging-driven workflows [[20], [45], [46], [47]].

AI-enhanced imaging augments CBCT and MRI quality through noise reduction and resolution enhancement, supporting clearer target and organ at risk (OAR) visualisation, as demonstrated using super-resolution neural networks in adaptive MRI workflows [48]. Improved image quality strengthens auto-segmentation performance and increases confidence for end users, including RTTs and radiation oncologists [49]. AI-accelerated image acquisition and reconstruction further reduce patient waiting time and intra-fraction motion [50], with evidence from diagnostic imaging supporting translation into radiotherapy workflows [[29], [51]]. These advantages in image quality and acquisition also support subsequent developments in synthetic CT generation and planning workflows.

Building on these imaging advances, AI-based synthetic CT generation offers a pathway to faster, dosimetrically accurate planning without additional imaging [52]. For synthetic CT to improve oART efficiency, reconstruction approaches must be sufficiently rapid, robust and uncertainty-aware [53], [54], [55], [56]. Beyond imaging, automated treatment planning could substantially reduce planning and console waiting time [57], [58], provided decision making, contour evaluation and QA time are not increased [59], [60].

In this context, emerging evidence indicates that oART is not required for every fraction [[39], [58], [61]]. Selective adaptation, applied only when dosimetric or clinical benefit is anticipated, can conserve replanning time, patient preparation time (e.g. bladder filling) and staff resources [39]. AI-based decision-support tools may assist in identifying these fractions, guiding adaptive frequency to optimise value [[10], [11], [50], [62]]. As also stated by Huynh et al. [50], AI might provide tools to predict which patients require adaptation of treatment and the ideal time point at which it should occur, or to identify the need to adapt treatment plans in order to maximise local tumour control and reduce side effects.

Extending beyond selective adaptation, AI in oART is shifting toward higher order workflow support. Emerging applications include prediction of patient motion and anatomical change, automated pre-adaptation preparation, real time quality metrics during delivery and threshold-based escalation prompts to involve physicians or physicists when increasing oversight is required. [[38], [63], [64], [65], [66], [67], [68], [69]] These developments shift AI from task acceleration toward clinical decision support, with direct implications for efficiency and value-based health care. Accordingly, safe implementation requires explicit governance. AI tools require transparent validation across diverse patient populations and clinical scenarios, with ongoing monitoring of performance and bias [57], [58], [64]. Human oversight remains essential for clinical accountability. Without structured training, credentialing and audit, AI implementation may increase variability rather than reduce it. Efficiency gains in oART therefore depend on deliberate integration of AI within professional frameworks and MDT decision-making structures, rather than automation alone [65], [66].

## Credentialing and Workforce Development for RTT-led oART.

While technological and workflow efficiencies are essential, their benefits depend on workforce capability. Without RTTs trained to independently manage adaptive workflows, including contour adaptation and plan approval, oART cannot operate sustainably at scale. RO light workflows can facilitate widespread integration of oART because the process is no longer dependant on a single physician, but instead is supported by a trained team of RTTs working within standard clinical practice [70]. Additionally, RTT led oART has been shown to be desirable both from a cost and efficiency perspective [71]. In this context, access, safety and quality are constrained by workforce readiness rather than technology.

Structured RTT credentialing provides a mechanism for translating technical capability into routine clinical practice, as outlined in established training and credentialing pathways. [21]. It supports safe expansion of oART by formally validating competence for defined adaptive responsibilities, reducing reliance on continuous physician presence. [[21], [24], [72]] oART represents both a technical and professional shift with adoption constrained by workforce bottlenecks and role dependency, particularly contour verification, plan assessment and adaptive treatment plan approval. RTT-led credentialing enables safe task reallocation in these domains, improving efficiency while advancing professional practice [[21], [73], [74]].

**Fig. 1:**
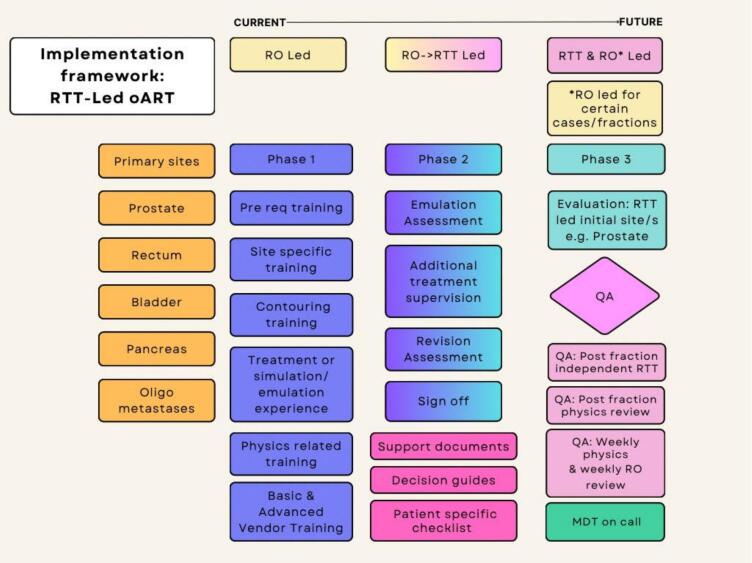
Operational pathway for RTT-led online adaptive radiotherapy.

Credentialing is the structured validation of competence [75], confirming that RTTs possess the education, training and demonstrated capability to manage adaptive decision points traditionally overseen by the MDT. Evidence shows credentialed RTTs can independently perform contouring [[33], [70], [71], [72], [73], [76], [77]] and plan review within structured governance models, including RTT-only adaptive delivery models in bladder oART [70]. Safety is maintained through clinician-lite oversight, post treatment QA and defined escalation protocols [21]. Importantly, RTTs demonstrate insight into their own training needs, supporting safe practice and ongoing development [[72], [78], [79], [80]]. The staged operational pathway is illustrated in Fig. 1. By reducing reliance on continuous physician involvement, credentialing improves access, operational efficiency, staff satisfaction and retention without compromising care quality [[11], [13], [72]]. While RTT credentialing does incur a cost, this is likely to be financially prudent overall and future research should evaluate this in greater detail. As oART becomes more widespread, training may be incorporated into undergraduate RTT programmes reducing the clinical department education burden.

International programs demonstrate both rigour and scalability, as reflected in national assessments of adaptive radiotherapy practice and workforce roles in Canada [81]. Multiple studies confirm that RTT-led oART is safe and efficient across disease sites on both MRI- and CBCT-guided adaptive platforms [[21], [33], [70], [71], [73], [76], [77], [78], [82], [83]]. A phased, MDT-aligned credentialing framework is recommended [21], culminating in live, independent MDT assessment across multiple patients to support shared ownership, evaluation and continuous refinement [82].

Ongoing skill maintenance is essential, particularly where efficiency gains depend on sustained clinical judgement and technical proficiency [21]. Credentialing frameworks must embed feedback mechanisms, periodic case and metric review, refresher training and contouring audits. Re-credentialing should be triggered by prolonged absence, software upgrades, reduced confidence or workflow change, ensuring safety and quality are maintained as oART programs evolve [84].

International experience demonstrates convergence toward common solutions, including RTT-led adaptive models under physician oversight, formal credentialing frameworks defining competencies and scope of practice, structured training programs that reduce variability in skill acquisition, and embedded MDT collaboration and governance structures. These interdependent solutions form the foundation of sustainable oART programs, supporting workforce development, AI integration and patient safety.

Implementation of RTT-led oART varies according to governance, regulation and training. In countries with established advanced practice frameworks and broader RTT scopes of practice, including the UK, Canada, Australia and several European nations, governance structures more readily support delegation of contour verification, adaptive decision making and plan approval within defined protocols [85]. This facilitates implementation of RTT-led workflows. Fig. 1 illustrates how evidenced-based training and credentialing operationalise the concept of ‘putting the RTT into RTT-led oART’. In contrast, jurisdictions where legislation restricts RTT scope of practice and reserves legal responsibility for adaptive decisions to clinicians or physicists face greater structural barriers, even when technology and workforce capacity are available [[21], [86], [87]].

Credentialing also acts as a professional lever, supporting advanced decision-making competencies and underpinning advanced practice radiation therapist (APRT) roles where appropriate [87]. APRT roles remain distinct and typically encompass leadership, governance and service-level responsibilities. Importantly, working to full scope within an oART workflow does not, in itself, equate to advanced practice and may, over time, align with standard practice within the profession. As adaptive cases increase in complexity, credentialed RTTs support delivery across a range of clinical scenarios, from prostate treatments to nodal boosting and small-volume OAR sparing. Without structured credentialing, RTTs risk being marginalised rather than driving technological progress and patient care [[88], [89]].

Challenges remain, including securing resources for training, navigating jurisdictional variation and addressing differing professional attitudes toward role reallocation and accountability. Empowering RTTs through credentialing supports the transition of from technological innovation to a sustainable, patient-centred standard of care, in which the professionals delivering it, RTTs, are as central as the platforms they use. However, without a shift toward an RO-lite framework, technical efforts to boost efficiency may be negated by a bottleneck at the point of delivery, limited by the RO availability on the treatment unit.

## Future Directions

Future efforts must address critical evidence gaps through multi-institutional studies evaluating workflow efficiency, the impact of AI integration and adaptive dose-outcome correlations to guide evidence-based patient selection [[23], [83]]. Global credentialing standards for RTTs would support workforce mobility while ensuring consistent QA across regions [[90], [91]].

Advocacy should prioritise equitable access to training, particularly in resource-constrained settings where educational gaps compound radiotherapy inequalities [[92], [93]]. Sustainable use of oART depends on workforce development that keeps pace with capital investment. [[89], [90]]

Strategic patient adaptation, alongside longitudinal training pathways and succession planning, is required to ensure adaptive platforms achieve sustained clinical use for patients most likely to benefit, rather than becoming under utilised due to preventable workforce limitations [[21], [94], [95]].

## Conclusion

**Fig. 2:**
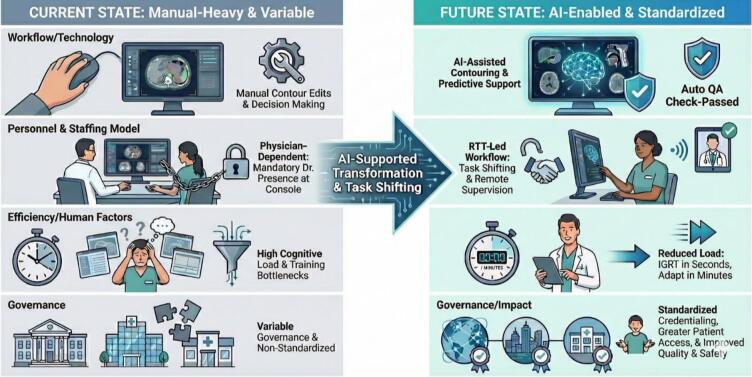
Transition from physician dependent adaptive radiotherapy to AI enabled RTT led workflow.

oART represents a significant evolution in radiotherapy delivery, offering clinical, operational and patient-centred benefit when implemented sustainably. This international collaboration highlights that RTT-led delivery models, supported by AI integration, structured training and credentialing, are essential to realising the full potential of oART (Fig. 2). Establishing RTT-led oART as standard practice requires formal recognition within the MDT, strengthened professional frameworks and ongoing evaluation of clinical and service outcomes. Strategic investment in workforce capacity, alongside a clear obligation for vendors to deliver clinically integrated AI solutions that reduce workflow burden, is essential to ensure equitable access to personalised oART across diverse global healthcare systems.

## Declaration of generative AI and AI-assisted technologies in the manuscript preparation process.

During the preparation of this work the authors used Google Gemini in order to generate the manuscript figures. After using this tool/service, the authors reviewed and edited the content as needed and takes full responsibility for the content of the published article.

## CRediT authorship contribution statement

**Meegan Shepherd:** Writing – review & editing, Writing – original draft, Visualization, Project administration, Investigation, Data curation, Conceptualization. **Bethany Williams:** Writing – review & editing, Writing – original draft, Visualization, Investigation, Data curation, Conceptualization. **Anna Dinkla:** Writing – review & editing, Writing – original draft, Visualization, Investigation, Conceptualization. **Brayden Geary:** Writing – review & editing, Writing – original draft, Visualization, Investigation, Data curation, Conceptualization. **Sarah Barrett:** Writing – review & editing, Writing – original draft, Visualization, Investigation, Conceptualization.

## Declaration of competing interest

The authors declare that they have no known competing financial interests or personal relationships that could have appeared to influence the work reported in this paper.
